# Delayed post-traumatic spinal cord infarction in an adult after minor head and neck trauma: a case report

**DOI:** 10.1186/1752-1947-6-314

**Published:** 2012-09-19

**Authors:** Viktor Bartanusz, Mateo Ziu, Leisha E Wood, Jean-Louis Caron

**Affiliations:** 1 Department of Neurosurgery, The University of Texas Health Science Center at San Antonio, 7703 Floyd Curl Drive, San Antonio, TX, 78229-3900, USA; 2 Travis County Medical Examiner’s Office, 1213 Sabine Street, Austin, TX, 78701, USA

**Keywords:** Spinal cord blood supply, Ischemia, Minor head trauma

## Abstract

**Introduction:**

Delayed post-traumatic spinal cord infarction is a devastating complication described in children. In adults, spinal cord ischemia after cardiovascular interventions, scoliosis correction, or profound hypotension has been reported in the literature. However, delayed spinal cord infarction after minor head trauma has not been described yet.

**Case presentation:**

We report the case of a 45-year-old Hispanic man who had a minor head trauma. He was admitted to our hospital because of paresthesias in his hands and neck pain. A radiological workup showed cervical spinal canal stenosis and chronic cervical spondylotic myelopathy. Twelve hours after admission, our patient became unresponsive and, despite full resuscitation efforts, died. The autopsy revealed spinal cord necrosis involving the entire cervical spinal cord and upper thoracic region.

**Conclusions:**

This case illustrates the extreme fragility of spinal cord hemodynamics in patients with chronic cervical spinal canal stenosis, in which any further perturbations, such as cervical hyperflexion related to a minor head injury, can have catastrophic consequences. Furthermore, the delayed onset of spinal cord infarction in this case shows that meticulous maintenance of blood pressure in the acute post-traumatic period is of paramount importance, even in patients with minimal post-traumatic symptoms.

## Introduction

The regulation of spinal cord blood flow (SCBF) in normal and pathological conditions is still largely unknown. Numerous experimental studies address the question of blood circulation in the injured spinal cord [1], but clinical cases of documented dysfunction of spinal cord circulation are of paramount importance for further understanding of this enigmatic phenomenon.

Delayed post-traumatic spinal cord infarction is a devastating complication described in children after injuries without vertebral fracture [2] or even after intensive physical exercise [3]. In adults, spinal cord ischemia has been reported to occur after aortic surgery, scoliosis correction, and profound arterial hypotension [4-6], but delayed spinal cord infarction after minor head trauma has not been described yet.

We report the case of a patient who was hospitalized for observation after sustaining a minor head trauma. Except for a few subjective symptoms in the upper and lower extremities, the results of a neurological exam were unremarkable. Twelve hours after admission, our patient developed a fatal infarction of the cervical and upper thoracic spinal cord. The goal of this case report is to point out the fragility of blood supply to the upper thoracic and cervical spinal cord in adults with chronic spinal canal stenosis, in which minimal hemodynamic perturbation may have catastrophic consequences.

## Case presentation

A 45-year-old Hispanic man was transported to our hospital after a blow with a dragline weight provoked head flexion. On presentation, he complained of lower-extremity numbness and pain in his back and around his abdomen. An examination revealed no point tenderness to palpation on his cervical, thoracic, or lumbar spine. The strength in the upper and lower extremities was preserved except for bilateral finger flexion weakness (motor grade 3). Hyporeflexia was noticed throughout. His rectal tone was normal. Hyperesthesia in a patchy distribution throughout his upper and lower extremities and patchy pinprick hypoesthesia in the lower extremities were observed. Proprioception was reduced only in his left toe.

**Figure 1:**
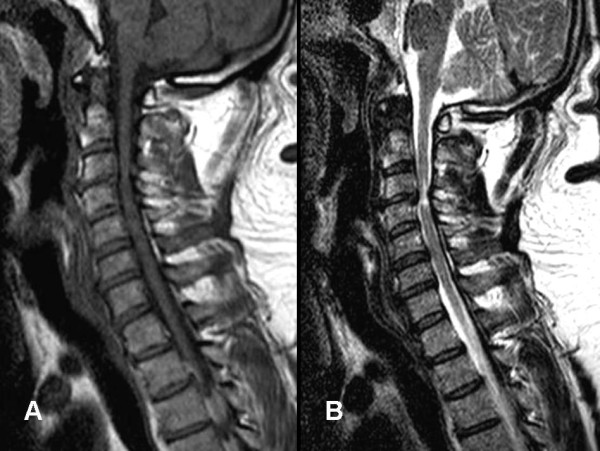
**Magnetic resonance imaging (MRI) of the cervical spine shows cervical spinal canal stenosis at the C3-4 segment. **(**A**) T1-weighted MRI image without contrast. (**B**) T2-weighted MRI image. The T2-weighed sagittal image shows intramedullary hyperintensity and no signs of spinal cord swelling. The images are compatible with chronic spondylotic cervical myelopathy.

A computed tomography scan of his cervical spine showed multilevel degenerative changes that were most severe at C3-4 but no fracture. Magnetic resonance imaging of his cervical spine demonstrated significant spondylotic spinal canal stenosis at the level of C3-4, increased intramedullary T2-weighted signal, and spinal cord atrophy at that level. The paraspinal soft tissues were within normal limits (Figure 1). The radiological picture was indicative of chronic spondylotic cervical myelopathy.

Because the clinical status of our patient was not worrisome, he was assigned to an intermediate hospital bed, and neurological and vital signs were monitored every 2 hours. On 2-hour vital sign controls, his systolic and diastolic blood pressures were above 120mmHg and above 70mmHg, respectively, throughout his stay and his heart rate was within the normal range of 70 to 85 beats per minute. Oxygen saturation was above 90% on 2 liters by nasal cannula. Throughout his hospital stay, our patient complained of intermittent subjective symptoms, such as paresthesias, whole-body pain, and fear of death, but the results of regular 2-hour exams did not show any worsening of the motor exam results. Approximately 8 hours after admission, a urinary catheter was introduced for increasing difficulty with urination. Two hours after placement of the urinary catheter, he was still stable. On the next 2-hour check (4 hours after the catheter placement), he was found in bed unresponsive. Immediate resuscitation was initiated but was not successful.

**Figure 2:**
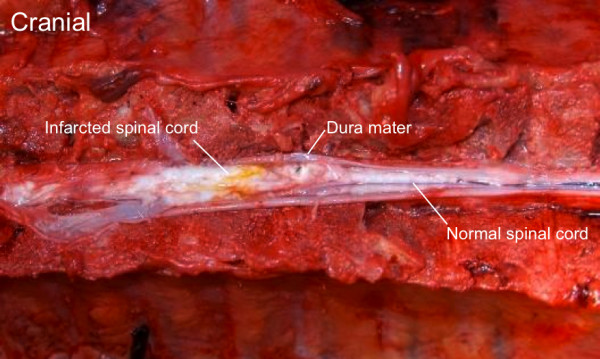
**Gross view of the anterior spinal cord in the cervical and thoracic spinal canal.** On the right side, a normal-appearing spinal cord is localized in the mid-thoracic region. To the left, the cranial portion of the spinal cord is localized from the mid-thoracic to the upper cervical region. A loss of normal structure and complete disintegration of the spinal cord on the left side of the picture indicate necrosis in the upper thoracic and entire cervical spinal cord.

The autopsy revealed macroscopic signs of spinal cord necrosis extending along the entire cervical spinal cord to the mid-thoracic region (Figure 2). The microscopic examination showed diffusely scattered large eosinophilic degenerating neurons in a background of disintegrating glial tissue consistent with ischemic necrosis.

## Discussion

Delayed spontaneous post-traumatic cervical spinal cord infarction in adults is a devastating condition that, to the best of our knowledge, has not been described yet. Cases of spinal cord ischemia after minor trauma are well documented in children [7,8]. Nance and Golomb [2] reviewed a series of children with spinal cord infarction without vertebral fracture and concluded that hypotension and fibrocartilaginous embolism were the principal etiological factors of spinal cord infarction after minor trauma or extensive physical exercise. Ischemia of the spinal cord is believed to be the underlying mechanism of spinal cord injury without radiographic abnormality [9,10].

The exact mechanism of delayed spinal cord infarction in our case remains unknown, but we speculate that premorbid degenerative spinal canal stenosis played an important role in this respect. The mid-cervical region with chronic cervical myelopathy at the C3-4 level seems to have represented a hemodynamically fragile territory. Experimental evidence in cats shows that vascular autoregulation is deranged even after mild spinal cord trauma leading to transitory hyperemia [11]. Experimental studies on rats demonstrated that mild spinal cord injury was followed by a transitory decrease in SCBF [12] and this decrease was paralleled by a transitory drop in mean arterial blood pressure (MABP) [13].

Given the results of these experimental studies, it is conceivable that the delayed spinal cord infarction in our patient was the end result of several pathophysiological processes. First, our patient withstood a mild head injury after being struck by a dragline weight. This blow to his head provoked hyperflexion of the cervical spine, which caused spinal cord compromise in the narrow cervical spinal canal, hence the mild sensory symptoms in the upper and lower extremities at admission. Second, this mild cervical spinal cord affliction (even if the clinical symptoms were minimal) had the further pathophysiological consequence of post-traumatic hyperemia because of perturbed vascular autoregulation. We hypothesize that the combination of locally decreased SCBF and transitory diminution of MABP during the night ultimately triggered infarction of the cervical spinal cord.

Vascular territory involvement is another important topic in the pathophysiology of spinal cord infarction. Pathological studies of infarcted human spinal cords indicate that infarctions occur most frequently in the most metabolically active lumbosacral and cervical segments [14]. This finding is contrary to the historical concept of ischemic vulnerability of the “watershed zone” centered at the mid-thoracic levels but is plausible in the context of the angiosome concept of blood supply to the spinal cord. Based on the angiosome concept, the arterial supply to the spinal cord follows a segmental, rather than longitudinal, distribution [15]. So, during arterial hypotension, the most vulnerable spinal cord segments are those that are the most active metabolically. The fact that our patient had an infarction to the cervical intumescence is in good agreement with the above findings. With respect to pial versus sulcal artery involvement, Ishizawa *et al*. [16], in a histopathological study of hemodynamic spinal cord infarction, showed that the infarction involved the gray matter supplied by the central artery while the periphery of the spinal cord supplied by the pial arteries remained intact. In our patient, the pathological finding was altered by the pre-existing cervical spondylotic myelopathy, and delineation between pial versus sulcal vascular territories was not possible.

## Conclusions

In this case report, we describe a catastrophic outcome of an adult admitted to our hospital after a minor head trauma with the clinical picture of chronic spinal canal stenosis. He had a cervical spinal cord infarction about 12 hours after admission. In retrospect, there were no alarming symptoms or signs that would have prompted us to suspect this imminent danger during his hospital stay. This case report highlights the complexity of spinal cord hemodynamics, especially when new acute injury is superimposed on a chronic pathological spinal cord condition. No diagnostic technique allows continuous monitoring of blood flow in different spinal cord regions. Therefore, it is impossible to envision any real-time therapeutic intervention. Meticulous maintenance of physiological MABP in the post-traumatic period remains the only preventive measure for avoiding delayed spinal cord infarction in these patients [17].

## Abbreviations

MABP: mean arterial blood pressure; SCBF: spinal cord blood flow.

## Consent

We attempted to obtain written informed consent for the deceased patient presented in the case report by trying to locate immediate family members. No valid contact information was available for living family members. Therefore, written informed consent could not be obtained from the patient's next of kin despite all reasonable attempts. The authors have taken careful effort to ensure that no patient-identifying information was placed within this manuscript.

## Competing interests

The authors declare that they have no competing interests.

## Authors’ contributions

VB and MZ analyzed and interpreted the patient data regarding the relationship between acute decline in SCBF, chronic cervical spondylotic myelopathy, and spinal cord infarction. LEW performed the histological examination. J-LC was a major contributor in writing the manuscript. All authors read and approved the final manuscript.
